# Comparing the incidence of postoperative painful bladder spasm between malecot catheter and 3-way Foley catheter: a clinical trial

**DOI:** 10.1097/MS9.0000000000001913

**Published:** 2024-03-18

**Authors:** Mojtaba Farahani, Keramat Dehghani, Parisa Shojaei

**Affiliations:** a General Practitioner, Faculty of Medicine, Tehran Medical Sciences, Islamic Azad University, Tehran, Iran; b Department of Urology, Faculty of Medicine, Tehran Medical Sciences, Islamic Azad University, Tehran, Iran; c Department of Community & Preventive Medicine, Faculty of Medicine, Tehran Medical Sciences, Islamic Azad University, Tehran, Iran; d Social Determinants of Health Research Center, Tehran Islamic Azad University of Medical Sciences,Tehran, Iran

**Keywords:** bladder spasm, hospital, open prostatectomy, pezzer catheter, Three-way foley catheter

## Abstract

**Background:**

In this study, the effect of Pezzer (as a Pezzer catheter) and three-way Foley catheters, used for suprapubic catheterization after open prostatectomy, was investigated in terms of early incidence of painful bladder spasms.

**Materials and methods:**

In this single-blind clinical trial study, 160 patients diagnosed with LUTS/BPH were placed in two groups of 80 Pezzer catheter (Pezzer(size 28)) and three-way Foley catheters (size 24) for suprapubic catheterization. The evaluated variables in this study are age, prostate weight, narcotics dose, Spasm frequency, International Prostate Symptom Score (IPSS), visual analogue scale (VAS), catheterization period.

**Results:**

The frequency of spasm in Foley group was significantly lower (4.4±7.26 vs. 6.28±4.4; *P* value=0.000). There was no significant difference between the two groups regarding the frequency of dysuria (*P* value=0.3).

**Conclusion:**

The findings of our study showed that despite slightly favourable results in using suprapubic Foley catheter compared to Pezzer catheters in patients with open prostatectomy in terms of pain and spasm frequency, there was no significant difference between the use of these two types of catheters.

## Introduction

Highlights

- Foley catheters are significantly more favourable in comparison to Pezzer catheters used for suprapubic catheterization in patients with open prostatectomy in terms of pain and spasm frequency.
- The prevalence of lower urinary tract symptom in men below forty years of age is low, but in men over eighty years, it becomes nearly 80%.
- Clinician removes clots by creating negative pressure with a syringe during bladder washing without worrying about the narrowing of the lumen.

Lower urinary tract symptom (LUTS) is defined by International Continence Society (ICS) as a symptom related to the lower urinary tract (frequency, urgency, nocturia, and dysuria). It may originate from the bladder, urethra, prostate (men) and/or adjacent pelvic floor or pelvic organs, or at times be referred from similarly innervated anatomy for example lower ureter^1^.

Indications for surgical treatment include LUTS, especially moderate-to-severe urinary symptoms associated with BPH and resistant to medical therapy, refractory urinary retention, recurrent urinary tract infection (UTI), recurrent gross haematuria, recurrent bladder stones, and bilateral hydronephrosis with impaired renal function^2–6^. For surgical treatment of LUTS/BPH, simple prostatectomy surgery is usually considered for people with large prostates in whom transurethral techniques are difficult to perform^7,8^. In both surgical methods, after the steps of accessing the prostate, removing the adenoma, and haemostasis manoeuvres, the surgical site is closed. At this stage, the bladder is probed with a Foley catheter through the urethra^9^. Complications related to the initial stage of insertion include cutaneous bleeding, bladder bleeding, and bladder trauma^10^. In later stages, the most common complication of bladder catheters is catheter-associated urinary tract infection (CAUTI). Urinary infection can lead to epididymitis or orchitis in men^11–15^. Many people around the world may experience bladder spasms. Not only does it cause contractions and pain in patients, but they also need to void their urine, which may be accompanied by leakage of urine^16^. This may have different effects on different activities of a person, such as sexual, social, family, and psychological, and it may cause dissatisfaction in everyday life. The best treatment for this spasm is to remove the urinary catheter^17^. The Pezzer catheter, with a slight difference in its structural shape, is a type of Pezzer catheter, which is mainly used for postoperative optimal drainage^18^. Our clinical investigations for postoperative care and follow-up during the hospitalization of patients who underwent simple open prostatectomy surgery in One of the hospitals of Iran show that the frequency of complaints of painful bladder spasms caused by Suprapubic catheterization is more common in cases with Pezzer catheter in comparison to three-way Foley catheter placement^19^. Also, doctors can have a very decisive role in treatment decisions by using clinical evidence resulting from research. In this regard, research was conducted by designing a clinical trial to compare the effect of Pezzer and three-way Foley catheters, used for suprapubic catheterization after open prostatectomy, on the early occurrence of painful bladder spasms.

## Methods

### Trial design

This study is designed as a clinical trial with a randomized control group, which was performed as a single-blind, single-centre study. The work has been reported as being in line with the CONSORT criteria.

### Participants

The study sample included patients diagnosed with LUTS/BPH who underwent simple open prostatectomy surgery.

### Sample size

Total of 160 cases in two groups of 80 were included in the study.

$$n=\frac{(Z_{1-\alpha /2}^{2}\times p\left(1-p\right)}{d^{2}}$$

According to a study in 2008 in Iran, 11.8% of people with benign prostatic hyperplasia needed open prostatectomy surgery^20^.

*P*=0.118 *d*=0.05

$$Z_{1-\alpha /2}^{2}=1.96$$

A total of 160 in two groups of 80 were included in the study.

### Randomization

The method of sampling and selection of samples for entering the study was non-random, available and consecutive among patients who were candidates for open prostatectomy surgery.

Inclusion criteria: Patients with severe complications due to prostate enlargement, including treatment-resistant urinary retention, persistent gross haematuria, bladder stones, recurrent UTI, evidence of upper or lower urinary tract dysfunction (such as azotemia/uraemia), and urinary incontinence (overflow/urge types) and with a prostate weight of more than 65–70 g (estimated by DRE and ultrasound examination) are candidates for open prostatectomy surgery and entering the study. Patients suspected of having prostate cancer (with increased PSA or abnormal DRE) can be candidates for open prostatectomy surgery and enroled in the study if the prostate biopsy results are negative.

For the allocation of samples to one of the trial groups (group A: intervention with Pezzer catheter) and (group B: intervention with three-way Foley catheter), the random method of simple block design and random selection of one of the six-block modes was used (AABB, ABBA, BBAA, BAAB, ABAB, and BABA). This method was used to complete the counting of the number required for the determined sample size. The allocation of the samples was done by someone outside the research group, and the surgeon was informed of this.

### Blinding

The design of this study is a clinical trial with a randomized control group, which was performed as a single-blind, single-centre study.

### Implementation

It includes the following items: Studying the clinical file and description of the patient’s surgery to find out the type of intervention performed (suprapubic catheterization with a Pezzer catheter (as pezzer catheter) vs three-way Foley catheter), demographic (age, etc.), clinical and laboratory data, Interview with the patient and physical examination to evaluate evidence related to painful bladder spasm, complete Bladder Spasm Symptom Scale questionnaire/checklist and the visual analogue scale (VAS). The investigated variables in this study are age, prostate weight, narcotics dose, Spasm frequency, International Prostate Symptom Score (IPSS), VAS, catheterization period.

**Figure 1:**
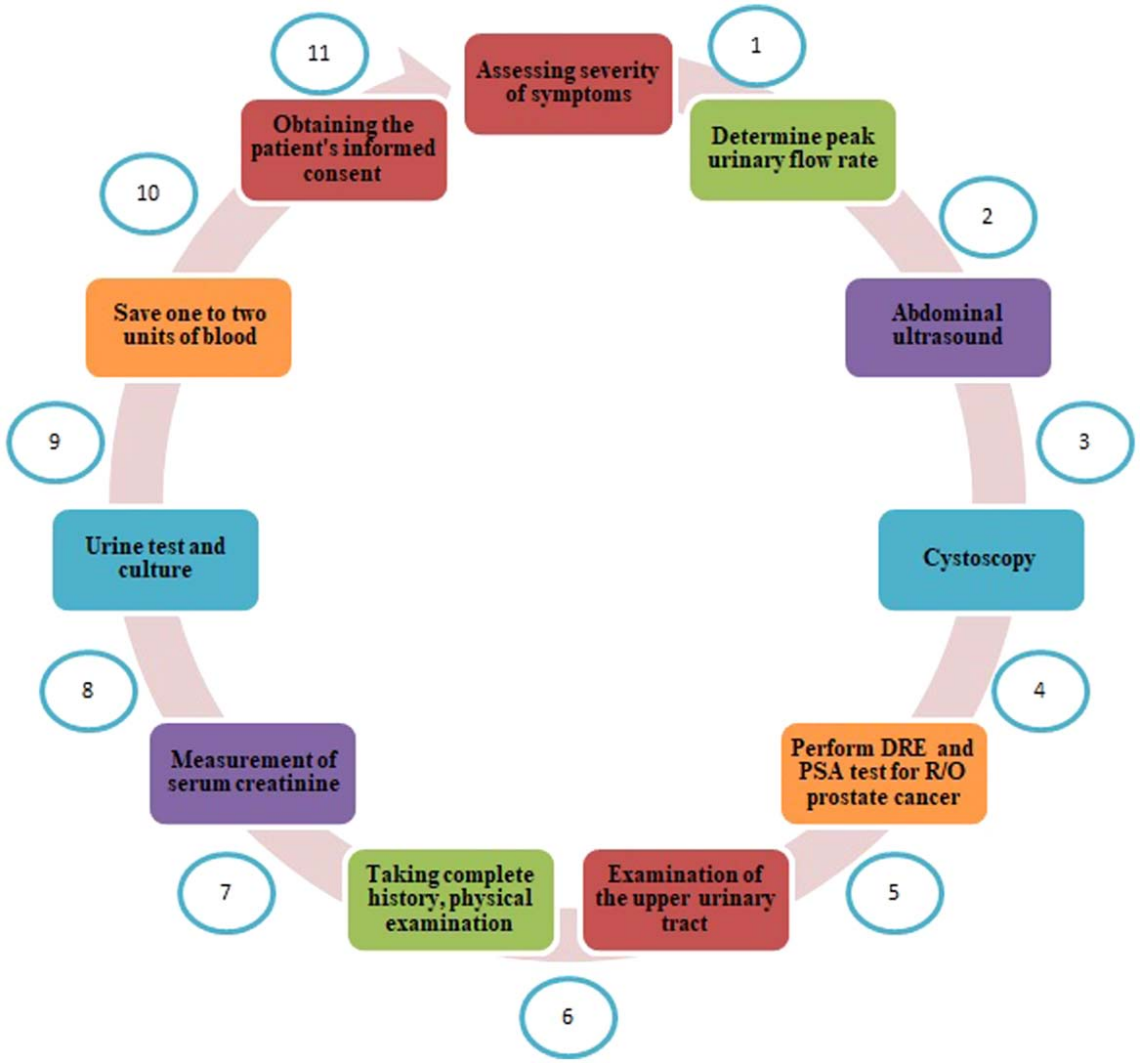
The following procedure before open prostatectomy surgery.

Before open prostatectomy surgery, the following examinations were performed for the study samples in all cases (Fig. 1): Complete examination of medical diseases by taking complete history, physical examination and laboratory tests, examination of the upper urinary tract in men with known renal disease, abnormal renal function, recurrent UTI or haematuria, Assessing the severity of symptoms with the IPSS scale “International Prostate Symptom Score, Uroflowmetry to determine peak urinary flow rate, ultrasound of residual urinary volume with abdominal ultrasound, cystoscopy for cases of haematuria, urethral stricture, stones or bladder diverticulum and confirming the presence of a large middle lobe or assessing the length of the prostatic urethra, Perform DRE and PSA test for R/O prostate cancer—if abnormal results, prostate biopsy was performed [tranrectal ultrasound (TRUS) biopsy was performed and due to lack of facilitations MRI was not done in all patients], Measurement of serum creatinine, urine test and culture, antibiotic therapy if there is evidence of UTI, Obtaining the patient’s informed consent regarding open simple prostatectomy and recording it in the hospital file after informing the patient about the advantages and disadvantages of this procedure.

Preparatory care for the day of surgery included the following: NPO from at least 8 h before the operation, Anaesthesia consultation for the urologist surgeon, the patient, and his family, administration of a dose of cephalosporin antibiotic, utilize compression devices in the lower limb to minimize the risk of deep vein thrombosis (if necessary), Save one to two units of blood

**Figure 2:**
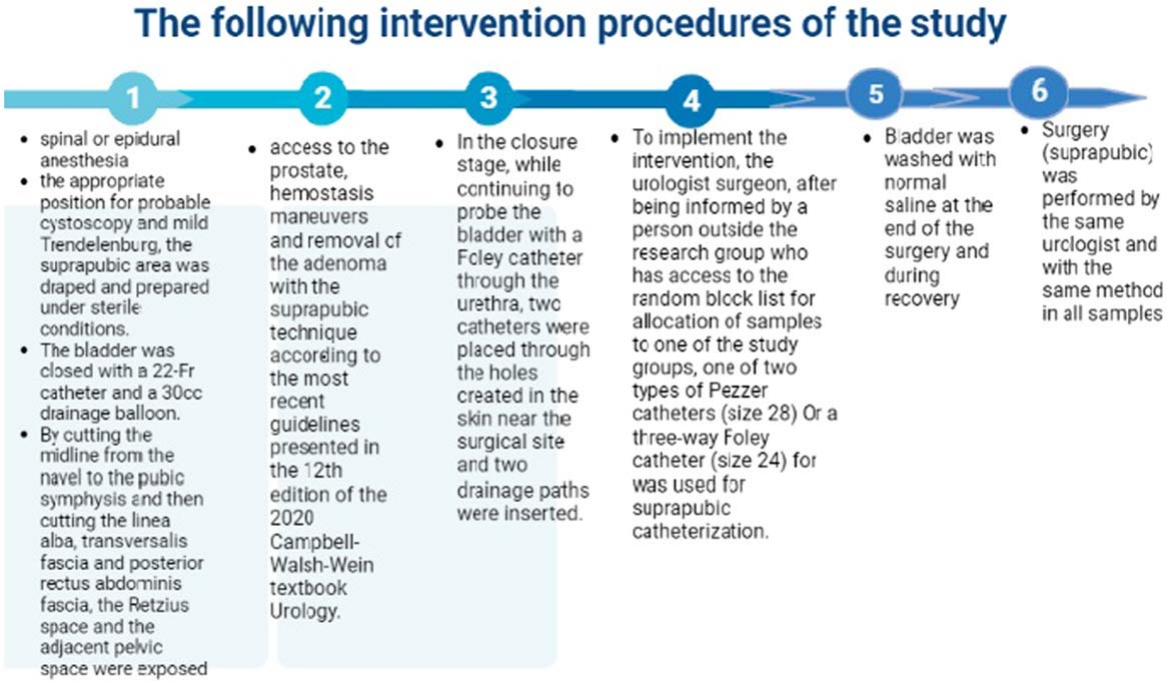
The following intervention procedure of the study.

The following procedures were done to perform the surgical technique and intervention of this clinical trial to insert the suprapubic catheter with one of the two Pezzer catheters or the three-way Foley catheter (Fig. 2): Usually, general anaesthesia is not performed and the preferred method is spinal or epidural anaesthesia, After inducing anaesthesia and placing the patient in the supine position, the appropriate position for probable cystoscopy and mild Trendelenburg, the suprapubic area was draped and prepared under sterile conditions. The bladder was closed with a 22-Fr catheter and a 30 ml drainage balloon. By cutting the midline from the navel to the pubic symphysis and then cutting the linea alba, transversalis fascia, and posterior rectus abdominis fascia, the Retzius space and the adjacent pelvic space were exposed. In the next step, access to the prostate, haemostasis manoeuvres, and removal of the adenoma with the suprapubic technique (prostatectomy through the bladder with an extraperitoneal incision in the lower anterior wall of the bladder) according to the most recent guidelines presented in the 12th edition of the 2020 Campbell-Walsh-Wein textbook Urology was done. Due to the surgeon’s experience, transvesical open prostatectomy was performed in all cases. In the closure stage, while continuing to probe the bladder with a Foley catheter through the urethra, two catheters were placed through the holes created in the skin near the surgical site, and two drainage paths were inserted. One tube is for drainage of the pelvic space, and the other is a suprapubic catheter. It enters the bladder directly from the wall of the bladder dome without entering the peritoneal space.

### Interventions

To implement the intervention, the urologist surgeon, after being informed by a person outside the research group who has access to the random block list for allocation of samples to one of the study groups, one of two types of Pezzer catheters (size 28) Or a three-way Foley catheter (size 24) for was used for suprapubic catheterization. The bladder was washed with normal saline at the end of the surgery and during recovery. Continuous washing of the bladder and drainage with a urethral catheter and suprapubic catheter was performed on the first day after surgery. The inserted catheters are not removed during postoperative hospitalization to monitor the secretions and the need for bladder washing. The pelvic drainage catheter is removed when the drain is clear, and the patient is usually discharged on the fifth day after surgery if there are no acute or serious complications. The suprapubic catheter is removed in the hospital between 5 and 7 days after surgery, and in the absence of any kind of complication, the Foley catheter is removed in the clinic on the 10–14th day. Surgery (suprapubic) was performed by the same urologist using the same method in all samples. All samples were under routine care after surgery and hospitalization.

### Outcomes

In order to evaluate the main outcome of the intervention, painful spasm of the bladder, the attending physician (a member of this trial research team) recorded the average sudden attacks of pain in the lower part of the abdomen and bladder on each day of hospitalization based on the questionnaire. The patient’s data were also collected about the sensation of the bladder bulge, the flow and colour of the urinary fluid in the urethral catheter, and the presence of burning or blood in the urine. Physical examination and observation in terms of urine leakage around the catheter and the colour of the urinary fluid were also done, and the relevant data were recorded in the data sheet (questionnaire). The data were adjusted according to the components of the Bladder Spasm Symptom Scale. According to Lu C, Wang H study in 2018^21^, scores were classified into the following 3 grades: (1) severe bladder spasm was defined when bladder spasms occurred once every several minutes, and patients kept holding their breath, accompanied by a feeling of urgent urination and severe lower abdominal pain, while the flushing fluid did not dribble, with increasingly darker colour and regurgitation; (2) moderate bladder spasm was defined when bladder spasms occurred once every 1 or 2 h, the flushing fluid did not dribble, with bloody urine spilling out around the catheter, along with paroxysmal abdominal pain and a sense of bladder bulge, but not severe; (3) mild bladder spasm was defined when bladder spasms occurred five times a day, with no bloody urine spilling out around the urinary catheter, with small changes in colour of the flushing fluid and absence of bladder spasms and the score and qualitative grouping of bladder spasm intensity were identified. Also, using a visual pain measurement scale (10 cm pain ruler), the patient was asked to indicate the intensity of bladder pain with one of the numbers from zero (no pain) to ten (worst possible pain). Other demographic and clinical variables, such as the age of the patient, the method of administration, and the dosage of painkillers, were recorded by studying the patient’s file.

### Statistical methods

SPSS software was used for statistical analysis. Categorized and qualitative variables such as the type of catheter used for suprapubic intubation and the qualitative grade of bladder spasm intensity were described with frequency and percentage index. For quantitative variables such as age, severity of bladder spasm with VAS scale, Bladder Spasm Symptom Scale questionnaire score, etc., first by using a histogram chart and drawing a normal curve and also performing the Kolmogorov–Smirnov test, the state of normal distribution of the data Determined. Then, the data with normal distribution were described with mean and standard deviation statistics, and the data with non-normal distribution were described with median and interquartile range statistics. independent *t*-test or Mann–Whitney U-Test were used to compare bladder spasm intensity score with VAS scale and bladder spasm questionnaire score between two study groups, depending on the normality of data distribution. *P* value less than 0.05 was considered to be statistically significant.

## Results

The mean age of the studied patients was 67.45±8.10 (Table 1). The two groups were not significantly different in terms of age, prostate weight and IPSS score, narcotics dose, and catheterization period (*P* value≥0.05). However, it was significant in the VAS variable, so the mean of the Foley group was 4.31 ± 1.10, and the Pezzer group was 5.11 ± 1.36. In the Spasm frequency, the mean of Foley group was lower than that of Pezzer, respectively, (6.28 ± 1.65, 7.26 ± 4.41). As the findings show, for both variables, the average was higher in the Pezzer group (*P* value≤0.05) (Table 2).

**Table 1:** Descriptive findings

variables	Mean	SD
Age	67.45	8.10
VAS	4.71	1.30
Prostate weight	60.73	6.63
Narcotics dose	10.50	9.47
Spasm frequency	6.77	3.35
Catheterization period	42.00	13.18

VAS, visual analogue scale.

**Table 2:** Mann–Whitney test; comparison of variables in the studied groups

	Group	Mean	SD	*P* (Mann–Whitney U)
Age	PEZZER	65.92	9.95	0.975
	FOLEY	65.80	10.84	
Prostate weight	PEZZER	61.38	6.39	0.242
	FOLEY	60.08	6.84	
IPSS	PEZZER	19.62	6.33	0.853
	FOLEY	19.20	5.30	
VAS	FOLEY	4.31	1.10	0.000*
	PEZZER	5.11	1.36	
Narcotics dose	FOLEY	10.43	8.86	0.792
	PEZZER	10.56	10.09	
Spasm frequency	FOLEY	6.28	1.65	0.000*
	PEZZER	7.26	4.41	
Catheterization period	FOLEY PEZZER	42.30 41.70	13.76 12.66	0.775

IPSS, International Prostate Symptom Score; VAS, visual analogue scale.

The χ^2^ test showed that painful bladder spasm, dysuria, urinary stones in the Foley and pezzer group are not significant in the two groups (Value≥0.05) (Table 3).

**Table 3:** Comparison of urinary stones, dysuria and painful spasm in the studied groups

	Group, *n* (%)		
	PEZZER	FOLEY	*P* (χ^2^)
Urinary stone			
+	19 (57.6)	14 (42.4)	0.217
−	61 (48.0)	66 (52.0)	
Painful bladder spasm			
Mild	7 (8.8)	15 (18.8)	0.140
Moderate	11 (13.8)	7 (8.8)	
severe	62 (77.5)	58 (72.5)	
Dysuria			
+	25 (54.3)	21 (45.7)	0.300
−	55 (48.2)	59 (51.8)	

## Discussion

The findings of our study showed that 75% of the studied patients experienced severe spasm, and 11.3% experienced moderate spasm following open prostatectomy surgery. The results showed the favourable state of bladder spasm in the Foley group; these differences are significant in comparison with Pezzer catheterization. The findings of the study by Yates and colleagues and Zhang and colleagues also showed that bladder spasm is common after urinary tract surgery, which has not consistent findings^22^. Krane *et al.* ^23^ stated that PST (percutaneous suprapubic drainage) provides adequate urinary drainage after RALP with less discomfort and no increased risk of urethral stricture. In the study of Orikasa and colleagues, out of 65 cases of total retropubic prostatectomy, 42 cases underwent suprapubic cystostomy, and Foley urethral catheter was used for 23 cases. In the cystostomy group, more than 85% and 69% of men, respectively, had no urinary symptoms during the catheterization period and no painful urination after catheter removal, while in the Foley group, 91% and 65% felt these symptoms. In our study, the average pain score in Foley and Pezzer groups was reported as 4.31±1.1 and 5.11±1.37, respectively, and showed a significant difference. Despite the higher average drug consumption in the Pezzer group, the differences were not significant. In Morgan’s study, a comparison with urethral catheter drainage and suprapubic tube after robot-assisted laparoscopic prostatectomy resulted in relatively better patient satisfaction, and no significant difference was observed between the groups in terms of bladder spasm, general pain, and complications reported by the patient^24^. Orikasa *et al.* ^25^ stated that according to these findings, prostatectomy without urethral catheter is a suitable alternative to prostatectomy with Foley catheter and improves the quality of life of patients in the days immediately after the operation. In the study by Prasad and colleagues, the researchers studied whether suprapubic catheter drainage with early removal of the urethral catheter compared to drainage with the urethral catheter alone caused pain relief in post-operation period. The primary analysis of the data obtained from the patients showed that the two groups did not differ in terms of the average VAS at any of the three time points of the study and in terms of the percentage of patients complaining about the catheter as the most common cause of discomfort—they were similar. Also, two groups had no significant difference in terms of treatment satisfaction^26^. In 2018, Galfanoand and colleagues’ research group conducted a prospective and non-randomized study with the aim of evaluating the difference between two different methods of urinary drainage, including the suprapubic tube (SPT) and standard urethral catheter (UC) in radical prostatectomy surgery with Robot-assisted and Retzius muscle-sparing (RS-RARP) in terms of discomfort, complications, and functional outcomes. The findings showed that the median postoperative pain score was similar in the two groups. No difference was found in terms of complications. These researchers concluded that a suprapubic tube is more convenient than a urethral catheter for urinary drainage after RARP. a possible advantage of SPT is fewer postoperative problems related to anastomosis^27^. The statistical findings of Li and colleagues showed that the pain after the RARP operation was less in the suprapubic catheter (SPC) group than in the transurethral catheter (TUC) group, both within three days and also within five days after the operation. In conclusion, these researchers stated that in cases of robot–assisted radical prostatectomy (RARP) surgery, patients in the SPC group suffer less from postoperative pain compared to the TUC group, and SPC can be a better alternative to TUC^28^. In the 2016 study by Martinschek and colleagues, the surgical outcome of catheter-related patient pain and discomfort was measured. In this regard, 62 patients undergoing RALP were prospectively randomized to urinary drainage with a urethral catheter (UC) or with a suprapubic tube (SPT). Functional results were assessed with standardized questionnaires (IPSS, IPSS Bother Score, IIEF and Visual Analogue Scale) preoperatively, after catheter removal, and 1 year after surgery. Moreover, bother by the catheter as well as pain due to the catheter, was assessed. In terms of personal hygiene, SPT was significantly more favourable than the other method. On days after surgery, the two groups did not have a significant difference in terms of pain scores. (Except for the 5th and 6th days when SPT was significantly more favourable). One year after surgery, there was no significant difference between the two groups regarding urinary function and IPSS^29^. Finally, the issue that comes up is the pain caused by the fear or rejection of the catheter, which can be treated after the cause of the pain is determined. If the cause is known, we can perform the necessary interventions before permanent damage occurs. In general, one of the serious side effects of indwelling urinary catheters is bladder spasm. For safe catheterization, training in its placement, on-site care and competence of caregivers, and knowledge of the type of urethral catheter is required.

## Limitations

Due to the lack of sufficient studies regarding the subject discussed in this research, it is suggested that larger studies be implemented by considering more variables in order to obtain more accurate results while checking the validity of the findings obtained from this study.

## Conclusion

A standard procedure performed by many health professionals is urethral catheterization, which is associated with many complications and medical malpractice. The findings obtained from our study showed that Foley probes are significantly more favourable than Pezzer probes used for suprapubic probes in patients with open prostatectomy in terms of pain and spasm frequency. However, there is a difference in terms of other factors. It was not observed between the groups.

## Ethics

The protocol of this research plan was presented to the ethics committee in hospital and university research for review and received the ethics code IR.IAU.TMU.REC.1399.508 and then it was registered in the system of the International Center for Registration of Clinical Trials of Iran (IRCT) with the code IRCT20220121053778N1.

The researchers of this research plan are obliged to comply with the codes of “General Ethics Guidelines for Research with Human Subjects in the Islamic Republic of Iran”, including:

- The health and safety of the patients during and after the research is the first priority.
- The design and implementation of the research is in accordance with accepted scientific principles based on current knowledge and based on a complete review of scientific sources.
- Necessary arrangements were made to help the patient if necessary and in emergency situations.
- Informed and freely written consent was obtained from the patients Before entering the study and in this regard, information was given to the patient, including: the title and objectives of the study, the duration of the study, the method to be used (including the possibility of random allocation to the group) intervention or control), the benefits and harms that the study is expected to include, the possibility of withdrawing from the study by personal decision and etc.
- The principle of confidentiality was respected and the patient’s secrets and privacy were preserved.
- The patients were assured that not accepting to participate in the trial or not continuing to cooperate has no effect on the medical services provided to the individual in the hospital.

## Consent

Written informed consent was obtained from the patient for publication and any accompanying images. A copy of the written consent is available for review by the Editor-in-Chief of this journal on request.

## Source of funding

Not applicable.

## Author contribution

Protocol/project development D.K., F.M., and S.H.P. Data collection or management D.K. and F.M. Data analysis S.H.P. and F.M. Manuscript writing/editing S.H.P. and F.M. All authors contributed to the conception, design and development of recommendations. All authors read and approved the final manuscript.

## Conflicts of interest disclosure

Not applicable.

## Research registration unique identifying number (UIN)

Registry used: Iranian Registry of Clinical Trials Unique Identifying number or registration ID: IRCT20220121053778N1 Hyperlink to your specific registration (must be publicly accessible and will be checked): C:\Users\darmangah20\Desktop\https.docx.

## Guarantor

Parisa Shojaei, Mojtaba Farahani.

## Data availability statement

All data generated or analyzed during this study are included in this published article.
